# A novel daily QA system for proton therapy

**DOI:** 10.1120/jacmp.v14i2.4058

**Published:** 2013-03-04

**Authors:** Xiaoning Ding, Yuanshui Zheng, Omar Zeidan, Anthony Mascia, Wen Hsi, Yixiu Kang, Eric Ramirez, Niek Schreuder, Ben Harris

**Affiliations:** ^1^ ProCure Proton Therapy Center Oklahoma City OK USA

**Keywords:** proton therapy, daily QA system, beam output, beam range, beam symmetry, image registration

## Abstract

We describe the design and use of a daily quality assurance (QA) system for proton therapy. The QA system is designed to check the overall readiness of proton therapy system consistently within certain reference tolerances by a home‐made QA device (the QA device). The QA device is comprised of a commercially available QA device, rf‐Daily QA 3, a home‐made acrylic phantom, a set of acrylic compensators with various thicknesses, and a mechanical indexing jig. The indexing jig indexes the rf‐Daily QA 3, as well as the acrylic phantom, onto the patient treatment couch. Embedded fiducial markers in the acrylic phantom are used to check X‐ray image quality and positioning alignment accuracy of the imaging system. The rf‐Daily QA 3 is used to check proton beam output, range and symmetry with one single beam delivery. We developed in‐house software to calculate beam range and symmetry, based on various ion chambers' readings inside the rf‐Daily QA 3. With a single setup and one beam irradiation, the QA system is employed to check couch movement, laser alignment, image registration, and reference proton beam characteristics. The simplicity and robustness of this QA system allows for a total QA time of less than 20 minutes per room. The system has been in use at three proton therapy centers since June 2009.

PACS numbers: 87.55.Qr, 87.53.Bn

## I. INTRODUCTION

One of the QA challenges in proton therapy systems is the lack of commercially available instruments designed specifically for proton beams. With increasing number of proton therapy facilities in the USA and worldwide, it is more prudent than ever to implement comprehensive, efficient, and standardized QA procedures to check system readiness every day before starting patient treatments. Some properties of proton beams do not have equivalence in photon therapy, which requires a novel approach to designing efficient QA device and the corresponding workflow. Based on recommendations by various task groups and other studies,^(^ ^1^ ^–^ ^5^ ^)^ our daily QA process tests the safety, image‐guided patient position system, and reference proton beam characteristics (output, range, and symmetry). Table 1 is a summary of our daily QA procedures and their corresponding tolerances. Specifically, the tests include laser check, couch movement, X‐ray imaging, image registration, proton beam output, proton beam range, and beam symmetry.

**Table 1:** List of daily QA tests performed at the ProCure Proton Therapy Center.

Category	Parameter	Tolerance
	Door interlock	functional
	Audio and video	functional
Safety	Proton beam on light	functional
	X‐ray beam on light	functional
	Couch movement	<1mm
Mechanical	Digital image panel position	<1mm
	Laser	<2mm
	Image registration	functional
Imaging	PPS Correction vector calculation	<1mm
	Proton beam output	<3%
Dosimetry	Proton beam range	<1.0 mm
	Proton beam symmetry	<3%

At the time of writing this manuscript, there was no dedicated commercially available daily QA device designed specifically for proton therapy. Therefore, during the system development process, we set our goals to achieve the following: (1) daily QA would be performed by radiation therapy technologists (RTTs); and (2) the total time to perform daily QA should be comparable to the photon clinic's morning QA procedures. There are two major challenges in achieving these goals. First, RTTs are typically not trained for using dosimetry equipments such as an ionization chamber, cable, and electrometer. Therefore, the QA device designed for therapists should come with simple user interface. Second, beam time for daily QA is limited, especially in multiroom facilities, because beams are delivered in a sequential fashion and only to one room at a time. Therefore, it is desirable to have as few beam‐deliver requests as possible during daily QA. To achieve the established goals, we had to develop our own daily QA device that would make it possible for therapists to perform all tests using one single device, with one simple setup, and a single proton beam delivery. To our best knowledge, our center is the first proton treatment center where RTTs perform daily QA.

## II. MATERIALS AND METHODS

### A. Procure proton therapy system

Our proton therapy center houses four treatment rooms, including one fixed horizontal beam room, two inclined (horizontal and 60° from horizontal) beam rooms, and one gantry room. An IBA Cyclotron (IBA, Louvain‐la‐Neuve, Belgium) is used to generate proton beams of 230 MeV. The highest proton beam energy may be downgraded to lower energy by an energy degrader, then transported to any of four treatment rooms. Currently, uniform scanning nozzles are employed in all four treatment rooms at our center. The proton beam entering the uniform nozzle will pass through a scatter and then range modulator wheel immediately downstream. The beam is scanned laterally and vertically by two scanning magnets in a pattern with a constant frequency to deliver a uniform dose for a rectangular scanning area. It then passes through the main and backup ionization chambers that monitor the proton dose, and an extractable snout which holds an aperture and a compensator. Three snouts are in current use at our center: snout10, snout18, and snout25, corresponding to a maximum field size of 10 cm, 18 cm, and 25 cm diameter circular field, respectively. Similar to a passive scattering nozzle, the aperture is used to conform the treatment target laterally, and the range compensator is used to conform the distal boundary of the treatment target. Each spread‐out Bragg peak (SOBP) is delivered layer by layer, with the most distal layer delivered first and shallower layers delivered later. More details on our system delivery were described by Zheng et al.^(^ ^6^ ^)^

### B. Mechanical jig

**Figure 1:**
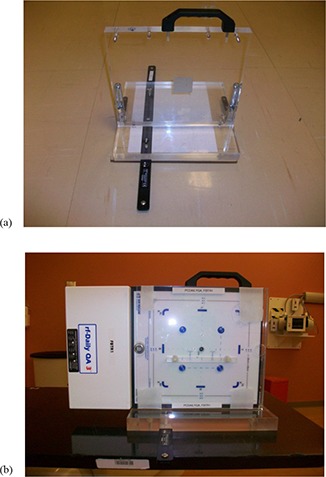
The mechanical jig (a) consists of two pieces of acrylic plates (2.6 cm thickness each). An index bar is attached onto the bottom plate and four metal screws are on the corners of the vertical plate to secure the rf‐Daily QA 3 device. The rf‐DailyQA3 (b) shown sandwiched between a 2.6 cm thickness acrylic plate and an acrylic imaging phantom. The central axis (CAX) chamber location is shown with an arrow and surrounded by four e‐Energy ion chambers (shown as blue circles at each corner of a square centered at the CAX chamber).

A mechanical jig was used for holding rf‐Daily QA 3 (Sun Nuclear Corporation, Melbourne, FL). The jig, as shown in Fig. 1(a), is comprised of two acrylic plates: a bottom plate and a vertical plate (2.6 cm thickness each). An index bar on the bottom plate of the jig is used for holding the jig onto the patient's couch. Four metal screws (¼ inch in diameter) on the corners of the vertical plate are used to tie rf‐Daily QA 3 onto the jig. The handle on the top of vertical plate is for carrying the device. Fig. 1(b) shows the jig holding rf‐Daily QA 3 and indexed onto patient couch and in the use position. An acrylic imaging phantom was attached to the front surface of the rf‐Daily QA 3. The phantom is used for tests of X‐ray imaging and image registration, which will be described in more detail in the Materials & Methods Section E below.

### C. Customization of the rf‐Daily QA 3 device

The rf‐Daily QA 3 is one of most widely‐used instruments of routine daily QA for photon linac machines, with a total of 13 built‐in ion chambers. Five $0.6\;{\text{cm}}^{3}$ ion chambers are used to measure photon output, flatness, axial symmetry, and transverse symmetry. Four ion chambers (e‐Energy ion chambers) with different inherent buildups are used to measure electron energy. All ion chambers are vented. Temperature and pressure are automatically measured by the instrument and applied to the readings. The instrument operates with Windows application software (Microsoft Corporation, Redmond, WA). Measurement data are collected and stored in a database for trend analysis, and can be exported to a text file for further analysis. Because rf‐Daily QA 3 is a wireless device with a built‐in rechargeable battery, no cable connection is needed. The wireless connection feature simplifies the setup process, as well as save QA equipment setup time.

In order to use rf‐Daily QA 3 for proton beam QA, customized modification is needed for both hardware and software. Out of the 13 built‐in ion chambers in rf‐Daily QA 3, we chose the central five for output test and verification of range and symmetry. The five ion chambers include the chamber at central axis (CAX), and four e‐Energy chambers ($e^{\text{TL}}$, $e^{\text{TR}}$, $e^{\text{BL}}$, and $e^{\text{BR}}$). The CAX is used for the output constancy test. It is placed approximately at the middle of the SOBP by a compensator with appropriate thickness. The reference reading for output is determined by using a calibrated ion chamber in water tank set at 1 cGy/MU for the reference beam. The reference condition point in our facility is set at the middle of SOBP for a beam of range 16 cm and modulation 10 cm. The four e‐Energy chambers are used for the verification of range and symmetry. They are physically located on the corners of an 8 cm square centered at the CAX chamber. A circular aperture, with a 10 cm diameter placed at 40 cm upstream from the isocenter plane provides a circular field projection of 12.5 cm diameter at the isocenter plane. The field size of 12.5 cm diameter is wide enough for irradiating all five ion chambers (CAX, $e^{\text{TL}}$, $e^{\text{TR}}$, $e^{\text{BL}}$, and $e^{\text{BR}}$) at the same time. Therefore, the output test and verification of range and symmetry can be performed by one beam delivery. Fig. 1(b) shows the detector locations and geometry. It is worth mentioning that our choice of using the 10 cm aperture for morning QA is simply a practical choice since patient treatments in all rooms are selected to start with the smallest field size. Therefore, there is no need to change the snout to accommodate larger field sizes after performing morning QA.

### D. Customized compensators

The proton distal range is defined by the point at distal falloff region where the proton dose is 90% of the maximum dose. To measure the distal range, a water tank scan is usually required. However, it is not practical to run water tank scan during daily QA due to time constraints. Instead, it is common practice to measure dose at several depths to monitor range fluctuation. These depths include at middle of SOBP, at the dose falloff region, and beyond the dose falloff region. We used the e‐Energy chambers ($e^{\text{TL}}$, $e^{\text{TR}}$, $e^{\text{BL}}$, and $e^{\text{BR}}$) in rf‐Daily QA 3 to check range fluctuation. These four chambers have different electronic buildups, which are $0.2\;g/{\text{cm}}^{2}$ for $e^{\text{TR}}$, $0.7\;g/{\text{cm}}^{2}$ for $e^{\text{BL}}$, $4.1\;g/{\text{cm}}^{2}$ for $e^{\text{TL}}$, and $4.3\;g/{\text{cm}}^{2}$ for $e^{\text{BR}}$. By using a compensator with appropriate thickness to reduce the beam energy, two chambers ($e^{\text{TR}}$ and $e^{\text{BL}}$) are set to measure the dose at close to the middle of SOBP, and another two ($e^{\text{TL}}$ and $e^{\text{BR}}$) for the dose at the falloff region. The charges collected by chambers $e^{\text{TR}}$ and $e^{\text{BL}}$, which are located at the flat portion of SOBP, are not sensitive to range fluctuation. However, the charges collected by chambers $e^{\text{TL}}$ and $e^{\text{BR}}$, which are located at the distal falloff region, are very sensitive to range fluctuation. They increase when beam energy is increased and decrease when beam energy is decreased. Therefore, the charge ratio of ($e^{\text{TL}}$ and $e^{\text{BR}}$) over ($e^{\text{TR}}$ and $e^{\text{BL}}$) is sensitive to range fluctuation. We used this ratio to monitor the daily fluctuation of proton beam range. We have performed a range sensitivity test to check on the sensitivity of the device to range fluctuations based on the charge ratio approach described above. We found that a slight change in the delivered range by one millimeter will cause a change in that ratio by roughly 30% using a beam with a range of 10 cm and modulation of 5 cm. This is due to the sharp dose gradient at the distal end of the beam. This test shows that our approach to determine the range using the rf‐Daily QA 3 is sensitive to range fluctuations.

**Figure 2:**
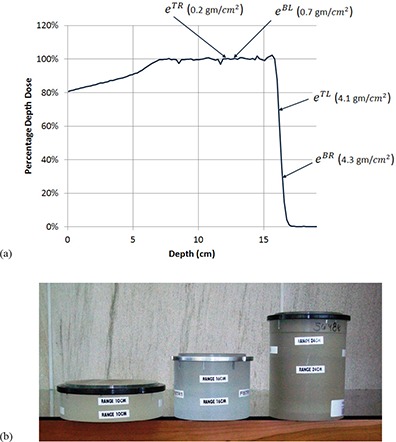
Approximate locations of the four chambers (a) on the PDD of a 16 cm range and 10 cm modulation proton beam. Acrylic compensators (b) used for various beam ranges: for range 10 cm (left), for 16 cm (middle), and for 24 cm range (right).

Figure 2(a) shows electron buildups of four e‐Energy chambers and their positions on the PDD of a proton beam with a range of 16 cm and a modulation of 10 cm. As shown in the figure, chambers $e^{\text{TL}}$ and $e^{\text{BR}}$ are used to collect charge at the distal falloff region, while chambers $e^{\text{TR}}$ and $e^{\text{BL}}$ are used to collect charge at the region close to the middle of SOBP. The compensator thickness depends on the beam energy. A high‐energy beam requires a thicker compensator. Figure 2(b) shows three compensators currently used in our facility for daily QA. The compensator on the left is designed for the beam of range 10 cm, the middle for range 16 cm, and the right for range 24 cm. The physical thicknesses of these compensators are 5.9 cm, 11.1 cm, and 18.0 cm, respectively. Although each treatment room uses one compensator, the availability of different thicknesses allows different rooms in the facility to check for different beam range values.

A symmetry test is usually not required during daily QA. We used the same device to measure beam symmetry by taking advantage of the fact that chambers $e^{\text{TR}}$ and $e^{\text{BL}}$ are physically located 11.3 cm apart from the central axis symmetrically. Since the water‐equivalent thickness $e^{\text{TR}}$ and $e^{\text{BL}}$ between is $0.5\;\text{gm}/{\text{cm}}^{2}$, the charge ratio from these two chambers is then used to monitor the beam symmetry. The ratio of them is close to 1.00. In fact, the baseline values of the ratio vary from 0.92 to 1.03 for our four treatment rooms. Strictly speaking, the charge ratio does not reflect the exact symmetry. However, it can be used to estimate the dose uniformity on the cross‐plane.

### E. Acrylic imaging phantom

**Figure 3:**
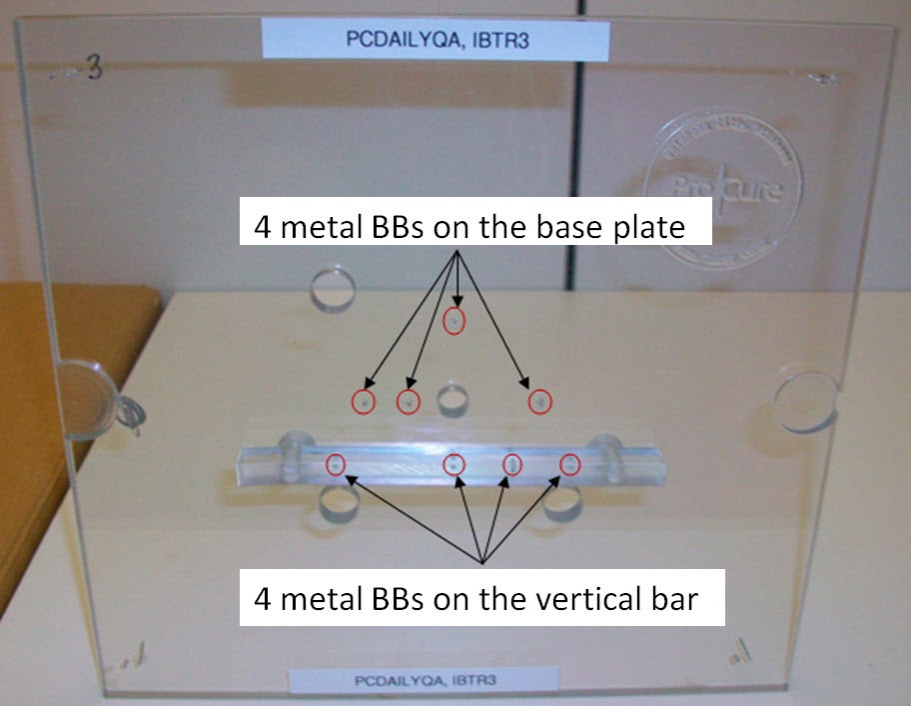
Location of implanted metallic BBs on the acrylic base plate facing the beam, and the attached vertical bar.

An acrylic phantom was attached to the front surface of rf‐Daily QA 3 for testing X‐ray imaging system functionality and image registration process. The acrylic phantom has eight metal BBs (1.44 mm in diameter) inserted. It is fastened to the front surface of rf‐Daily QA 3 by four metal pins which are inserted into four corresponding holes on the front face of rf‐Daily QA 3. The acrylic phantom consists of an acrylic base plate and an acrylic vertical bar. Both the base plate and the vertical bar have four metal BBs inserted. The dimension of the base plate is 26.3 cm×25.5 cm×0.9 cm. The dimension of the vertical plate is 1.5 cm×15.0 cm×0.9 cm. The bar is vertically mounted on the plate by two plastic screws. All four metal BBs on the base plate are visible on horizontal radiography. However, they cannot be seen clearly on the vertical radiography because the plate has a thickness of 25.5 cm along the vertical direction. Therefore, a vertical acrylic bar with another four metal BBs is inserted to allow for vertical radiography. Figure 3 shows the acrylic base plate and the vertical bar with four metal BBs implanted in each.

### F. daily QA procedure

Before the customized acrylic phantom was attached on the rf‐Daily QA 3, it was scanned at fine resolution (0.625 mm) in our CT scanner (GE LightSpeed RT16; GE Healthcare, Waukesha, WI) and a treatment plan was created using the CMS XiO treatment planning system (Elekta AB, Stockholm, Sweden). The treatment plan contains proton beam parameters (beam angle, range, and SOBP), as well as image dataset information (CT scan and DRR). The plan was then transferred to our oncology information system MOSAIQ (Elekta AB, Stockholm, Sweden) with room‐specific site setup and QA treatment fields.

The first test during our daily QA is the mechanical test of patient couch movement. Imaging inside the treatment rooms is achieved through the use of two orthogonal amorphous silicon panels (30 cm×40 cm). Using the imaging system, the morning QA device is registered to its reference image with a known shift from isocenter. The expected shift from the registration of the initial position of the device on the treatment couch has non‐zero values in X, Y, Z Cartesian coordinates, but expected to be reproducible to within 1 mm day‐to‐day. While performing daily QA, the QA device is attached onto patient couch at the predefined position by the index bar. The couch is then moved to a predefined position (setup position) in MOSAIQ. The match of wall laser projection to the crosshair label, which is on the back of the QA device, is used to verify device placement on the couch in addition to daily laser positional accuracy. The mechanical arm holding the X‐ray imaging panels is then moved to the imaging position. The wall laser projection should match a crosshair on the back of the horizontal X‐ray imaging panel as verified by eye. Since the width of crosshairs at isocenter is roughly 1 mm, any small positional deviation from a reference crosshair can be easily detected by human eyes.

Two orthogonal radiographs are then taken, one in the plane perpendicular to the proton beam axis and the other in a plane parallel to the beam axis. The base plate and vertical BBs are used as surrogates for the alignment process in the planes perpendicular and parallel to the beam axis, respectively. When all the BBs are properly registered to their expected location in the digitally reconstructed radiographs (DRRs), the correction vector (CV) for couch shift is calculated. After shifting the couch to the new position defined by the CV, two more radiographies are taken to verify the match between DRRs and radiographs. The CV is compared with baseline value and saved in MOSAIQ as a permanent record. The tolerance for the variation of CV is 2 mm. The comparison of CV with baseline value is mainly used for verifying the positions of X‐ray imaging panels. The imaging panels are mounted on a movable mechanical arm. They are extended to obtain X‐ray images during patient setup and retracted during proton beam delivery. If the imaging panels are not in the right position, the correction vector value will be different from the baseline value.

After the correction vector is applied to patient couch, the device should be in a position such that the center of CAX camber is at isocenter. A compensator with appropriate thickness is mounted to the snout. As mentioned in the previous section, the compensator thickness is chosen to place the chamber of CAX, $e^{\text{BL}}$, and $e^{\text{TR}}$ at depths close to the middle of SOBP, and ion chambers of $e^{\text{TL}}$ and $e^{\text{BR}}$ at the distal falloff region at the same time. A fixed number of MUs are then delivered to the QA device. Since the field projection at isocenter is 12.5 cm circular field, all five ion chambers (CAX, $e^{\text{TL}}$, $e^{\text{TR}}$, $e^{\text{BL}}$, and $e^{\text{BR}}$) are irradiated at the same time. The charges collected by five chambers in one beam delivery are used to monitor the variation of beam output, range, and symmetry. The charge collected by CAX is used for the output test. The charge collected by CAX is corrected automatically by temperature and pressure, and compared with the baseline value for the variation of output.

As mentioned previously, we designed compensators of certain water‐equivalent thicknesses so that the two ion chambers ($e^{\text{TR}}$ and $e^{\text{BL}}$) collected charge at depths close to the middle of SOBP and the other two ($e^{\text{TL}}$ and $e^{\text{BR}}$) at the distal falloff region. The charge ratio between the one collected at distal falloff region over the one close to middle of SOBP is then used to monitor the range variation. The thickness of compensator is chosen to make the charge sum from $e^{\text{TL}}$ and $e^{\text{BR}}$ to be around half sum of $e^{\text{TR}}$ and $e^{\text{BL}}$.

Therefore, one particular range requires one compensator with particular thickness, as shown in Fig. 2(b). Higher energy beam requires thicker compensator. Once the thickness of the compensator is determined, a charge ratio lookup table is constructed by measuring the charge ratios for four other ranges, by adjusting the beam energy to nominal range plus 1 mm, plus 2 mm, minus 1 mm, and minus 2 mm. By using the lookup table, the range variation can be determined from the measured charge ratio during daily QA.

## III. RESULTS

### A. Patient positioning system verification

**Figure 4:**
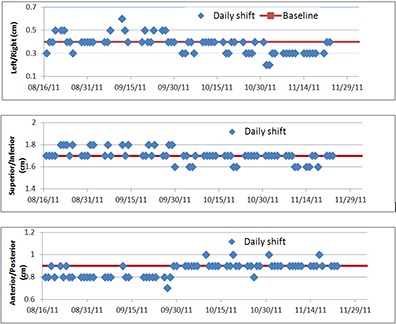
A three‐month trend for the correction vector (CV) of one treatment room. The CV includes translational shifts in three directions, left/right, superior/inferior, and anterior/posterior.

The correction vector temporal trends for a period of two‐and‐a‐half months in three directions in one of our treatment rooms are shown in Fig. 4. The red lines show the value of baseline vector. Daily variations in CV are well within 2 mm. If the CV daily value is greater than 2 mm in any direction from baseline value, a Winston‐Lutz test will be performed by a physicist immediately to verify the imaging isocenter is coinciding with the proton isocenter.

### B. Output check

**Figure 5:**
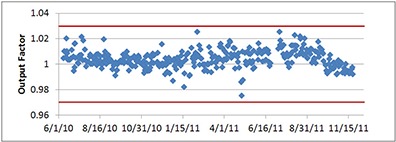
Output factor trend analysis from one of our inclined treatment rooms. The daily output fluctuation is usually within ±2%. The tolerance level (two red lines) is ±3%.

Daily proton output (cGy/MU) trend for one treatment room from June 2010 to Nov. 2011 is shown in Fig. 5. The action level of ±3% tolerance is represented by two red straight lines. As shown in Fig. 5, the output factor fluctuated within 2% most of time. In case the ±3% tolerance is exceeded, the output measurement will be performed again. If the tolerance is consistently exceeded, a solid water phantom with a thimble chamber will be used to perform the output measurement. The output factor is also cross‐checked by ion chamber measurement in water tank monthly. The results from water tank measurement and the QA device agreed well within 2%.

### C. Range check

**Figure 6:**
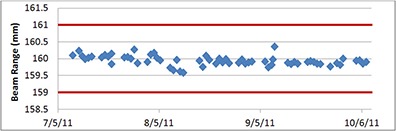
Range verification data for a period of three months in one treatment room. Most of the range fluctuation is within 0.2 mm and no fluctuation more than 0.5 mm is observed. The tolerance is 1 mm (two red straight lines).

Our facility is equipped with four treatment rooms. During daily QA, three beam ranges, 10 cm, 16 cm, and 24 cm, were chosen to be monitored. Figure 6 shows a trend analysis of the range variation in one of our treatment rooms. A proton beam of 16 cm range is chosen to be monitored for this treatment room. As shown in Fig. 6, the fluctuation of beam range is usually within 0.5 mm. The range tolerance is 1 mm in our facility, which is represented by two red straight lines. The fluctuation of measured proton range for 10 cm and 24 cm range beams that are used in other rooms is roughly in the same magnitude. In fact, we have seldom observed any fluctuation that is larger than 0.5 mm since we started monitoring the range verification in June 2011.

### D. Symmetry check

**Figure 7:**
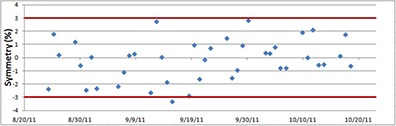
Beam symmetry data for a period of two months in one treatment room. Most of the data are within ±3% of the baseline value.

Symmetry values over a period of two months for one of our treatment rooms are shown in Fig. 7. The fluctuation of symmetry is within 3% most of the time. Occasionally, symmetry values may exceed 3% during the morning QA, but the majority of our data are well within the widely accepted 3% tolerance value. A comprehensive check on symmetry is performed during monthly and annual QA using a high‐resolution 2D array.

## IV. DISCUSSION

The daily QA system that we have developed and implemented in our clinic was designed to check with high accuracy for consistent output of certain beam and equipment parameters on a daily basis. A comprehensive set of checks can be performed using a combination of a commercially available rf‐Daily QA 3, an in‐house acrylic phantom, and a blank acrylic compensator of various thicknesses. The QA system has been employed at our center since June, 2010 and is currently in use in three proton therapy centers. All QA tasks are performed by radiation therapists and reviewed by the medical physicist on duty. Due to the simplicity of the setup and the associated data acquisition software, the overall QA time is less than 20 minutes per room. The integrity of the data is validated by comparing it against the accumulated measurements, as well as monthly QA measurements. Although there are differences among various proton facilities in regard to beam delivery system and associated hardware, we believe that our QA system approach can be adapted for any proton vendor system. Our tests are comparable to those used in other proton treatment facilities. We check interlocks, proton beam‐on lights, X‐ray beam‐on light, audio, and video for safety. We also check for laser positional accuracy and patient couch movement accuracy. In addition, we check X‐ray image quality, image registration, and correction‐vector calculation. We also check proton beam output, range, and symmetry for dosimetry.

Our QA practice currently does not require verification of SOBP ${\text{width}}^{4}$ daily since this is done accurately using high‐resolution one‐dimensional PDD measurements during monthly QA. The maximum buildup difference among electron energy chambers in rf‐Daily QA 3 is $4.1\;g/{\text{cm}}^{2}$. Currently we use flat‐top compensators to pull proton beam back uniformly. By this uniformly pull‐back approach, the buildup difference should be at least $6.0\;g/{\text{cm}}^{2}$ or larger, in order to measure proton beam with SOBP larger than 5 cm.

Arjomandy et al.^(^ ^5^ ^)^ investigated the use of a high‐resolution 2D ion chamber array for periodic proton beam QA and found that it was appropriate for checking proton beam flatness and symmetry, as well as dose output. However, considering factors such as cost, end‐user training, equipment setup time, and complexity of data interpretation, we believe that high‐resolution 2D arrays are more suitable for monthly and annual QA rather than daily QA.

## V. CONCLUSIONS

We developed a morning daily QA system that can be used to check various aspects of proton therapy room readiness. The system is comprised of a mechanical jig, a commercially available rf‐Daily QA 3, an acrylic phantom with implanted fiducials which is attached onto the rf‐Daily QA 3 device front surface, and a blank acrylic compensator of various thicknesses, as well as software for analysis of acquired measured data. Using this system, all major QA items are tested including patient couch movement, X‐ray imaging panel position, X‐ray image quality, image registration, dose output factor, beam range, and symmetry. The system is currently employed routinely at three proton therapy centers. It takes nearly 20 minutes for RTTs to perform daily QA per room. The system has been proven to be robust, efficient, and user‐friendly.
